# A dynamic interpretation of κFLC index for the diagnosis of multiple sclerosis: a change of perspective

**DOI:** 10.1007/s00415-023-11952-3

**Published:** 2023-08-28

**Authors:** Simona Toscano, Clara Grazia Chisari, Salvatore Lo Fermo, Giuseppa Gulino, Mario Zappia, Francesco Patti

**Affiliations:** 1 Department of Biomedical and Biotechnological Sciences, University of Catania, Via Santa Sofia 97, 95123 Catania, Italy; 2 Multiple Sclerosis Unit, University-Hospital G. Rodolico-San Marco, Via Santa Sofia 78, 95123 Catania, Italy; 3 Department “GF Ingrassia”, Section Neuroscience, University of Catania, Via Santa Sofia 87, 95123 Catania, Italy; 4 Central Laboratory, A.O.U. Policlinico-San Marco, Via Santa Sofia 78, 95123 Catania, Italy

**Keywords:** Multiple sclerosis, Diagnosis, κ free light chain, Case–control study, Biomarkers, Oligoclonal bands

## Abstract

**Background:**

Previous studies attempted to define the best threshold for κ free light chains (κFLC) index, confirming higher sensitivity (Se) but less specificity (Sp) compared with IgG oligoclonal bands (OCB) for the diagnosis of MS.

**Objective:**

To evaluate the diagnostic accuracy of different κFLC index intervals in a miscellaneous cohort of neurological patients, proposing a procedural flowchart for MS diagnosis.

**Methods:**

We analyzed data from 607 patients diagnosed with MS (179), CIS (116), other inflammatory (94) or non-inflammatory neurological diseases (218). Measures of diagnostic accuracy were reported for different potential thresholds of κFLC index, and for IgG OCB and IgG index. Binary logistic regression was to used to calculate the odds of being diagnosed with MS based on each increase of κFLC index.

**Results:**

CSF IgG OCB showed 72.2% Se (CI 95% 68.4–75.7) and 95.2% Sp (CI 95% 93.1–96.7) in discriminating between MS/CIS and controls, with an AUC of 0.84 (CI 95% 0.80–0.87). The highest diagnostic accuracy was reported for κFLC index cut-off of 5.0 (Se = 85.4%, Sp = 90.4%, AUC = 0.88), while a threshold of 11.0 exhibited higher Sp (95.5%, 95% CI 93.1–97.1) than IgG OCB. AUCs for all thresholds between 4.25 and 6.6 were not significantly different from each other, but were significantly higher than the AUC of IgG OCB (*p* < 0.05). The odds of being diagnosed with MS/CIS increased by 17.1% for each unit increase of κFLC index (OR = 1.17; 95% CI 1.12–1.23; *p* < 0.001).

**Conclusion:**

κFLC index performed better than CSF IgG OCB in supporting the diagnosis of MS/CIS, with the advantage of being a cost-effective and quantitative analysis.

## Introduction

CSF κ free light chains (κFLC) and the resulting κFLC index, calculated as the ratio between CSF/serum κFLC and albumin quotient, have been explored for years as an expression of the intrathecal humoral activity of plasma cells and a diagnostic biomarker for multiple sclerosis (MS) [1–3]. Several studies supported the high diagnostic accuracy of κFLC index, even when compared with CSF IgG oligoclonal bands (OCB), whose use in clinical practice as a diagnostic biomarker for MS relies on a strong level of evidence [4, 5]. Particularly, κFLC index has shown a higher sensitivity (Se) but a less specificity (Sp) compared with CSF IgG OCB in discriminating between MS and other neurological diseases [3, 6–11]. Noteworthy, κFLC index proved to be increased in up to 25% of MS patients with no evidence of CSF IgG OCB, who represent almost 5% of MS [7, 12, 13]. However, a recent meta-analysis highlighted no significant differences between these biomarkers in terms of diagnostic accuracy [14].

Different potential thresholds have been identified for κFLC index in literature, ranging from 4.25 [7] to 12.3 [6], representing a main limitation in comparing results from different studies. Since other inflammatory diseases of the central nervous system (CNS) can be characterized by a certain amount of intrathecal synthesis [15], the choice of low cut-off values, though maximizing sensitivity, is not suitable to distinguish between MS and other mimics [16]. Moreover, the proposal to use two different κFLC index thresholds to distinguish MS from inflammatory or non-inflammatory diseases [3] is reasonable but difficult to implement in clinical practice, since CSF analysis is often required precisely to clarify the potential inflammatory nature of neurological symptoms.

It could be argued that the interpretation of κFLC index as a dichotomous variable, by choosing a rigid threshold, is likely to minimize the potentialities of this diagnostic biomarker, which has the inherent advantage of being a quantitative measure in contrast with the detection of CSF IgG OCB, which is based on a qualitative analysis. Possibly, a more dynamic interpretation of κFLC index, relying on a risk stratification or identification of different value ranges, can allow clinicians to restrict the use of CSF IgG OCB analysis to fewer cases, thus saving time, reducing costs and assuring an operator-independent evaluation.

For this purpose, we evaluated the diagnostic accuracy of CSF IgG OCB, IgG index, and different cut-off values of κFLC index in a miscellaneous cohort of neurological patients, finally proposing a diagnostic procedural flowchart for the diagnosis of MS.

## Patients and methods

### Study population

We consecutively enrolled 607 patients admitted to the Neurology Clinic of the University Hospital “Policlinico G. Rodolico” of Catania, who underwent a diagnostic lumbar puncture (LP) in the period between 1st January 2017 and 7th February 2022. Patients were classified according to the diagnosis into four groups: MS, CIS, inflammatory neurological diseases other than CIS or MS (OIND), not inflammatory neurological diseases (NIND). MS and CIS were diagnosed according to the 2010 revision of McDonald’s criteria [17]. The study was approved by our local ethical committee. All patients signed a written informed consent before the execution of LP to authorize the procedure and to allow data collection and use for study purpose.

### Cerebrospinal fluid and serum samples collection and analysis

All patients underwent LP and venipuncture as part of their diagnostic workup. LP were performed at the bedside, using 25 Gauge atraumatic needles whenever possible, or 22 Gauge needles otherwise. For each patient, 2 mL of cerebrospinal fluid (CSF) divided into 0.5 mL aliquots and a serum 0.5 mL aliquot were collected in sterile polypropylene tubes and sent to the Central Laboratory of our University Hospital to be analyzed. CSF and serum paired samples were analyzed to determine κFLC index, IgG index, and CSF IgG OCB.

κFLC index was determined using an automated nephelometric immunoassay (Freelite LK016, The Binding Site Group Ltd). Monoclonal antibodies were used for the detection of FLC in serum and CSF. A 1:300 dilution was used for serum, while CSF was not diluted by default, but progressively increasing dilutions were used for progressively higher IgG concentrations (only for IgG > 5.0 mg/dL). κFLC index was calculated as the ratio between κFLC CSF/serum quotient (QκFLC) and albumin CSF/serum quotient (Qalb).

IgG index was calculated as the ratio between CSF/serum IgG corrected for Qalb, determined by nephelometry. We considered a threshold of 0.7, which is the most often used cut-off in clinical practice [18, 19].

CSF IgG OCB were detected by agarose gel isoelectric-focusing immunoassay (IEF) followed by immunoblotting (Helena Biosciences SAS IgG IEF kit), considering the presence of patterns 2 (≥ 2 IgG OCB bands in CSF) or 3 (IgG OCB bands in CSF and serum with at least 2 additional bands in CSF) as positive results[20].

### Statistical analysis

Data were analyzed with SPSS© (IBM Corp. IBM SPSS Statistics for Windows, Version 26.0). After assessed for normality with the Kolmogorov–Smirnov test, median and interquartile range (IQR) were provided for not normally distributed continuous variables. The Mann–Whitney *U* test (*U*) was used to compare medians between groups. Categorical variables were reported as frequencies and percentages. Chi-square test (*χ*^2^) and Cramer’s phi (*φ*) coefficient were used to compare categorical variables distributions among groups. Se, Sp, positive predictive value (PPV), and negative predictive value (NPV) were calculated for each biomarker. The area under the curve (AUC) of the receiver operator characteristic (ROC) curve was calculated to assess the diagnostic accuracy of the biomarkers. A *z*-test was used to compare AUCs of different κFLC index values and IgG OCB in a paired design[21].

Youden’s index was calculated for the chosen cut-off values for each biomarker and for other cut-off values tested in other studies, using the formula *J* = Se + Sp − 1. The point-biserial correlation coefficient (*r*_pb_) was used to measure the association between continuous and dichotomous variables. Binary logistic regression was used to analyze the relationship between κFLC index and the probability of being diagnosed with MS/CIS, with IgG index and IgG OCB as covariates. A *p* value of < 0*.*05 was considered significant for all tests, which were two sided.

## Results

### Patients’ characteristics

**Fig. 1:**
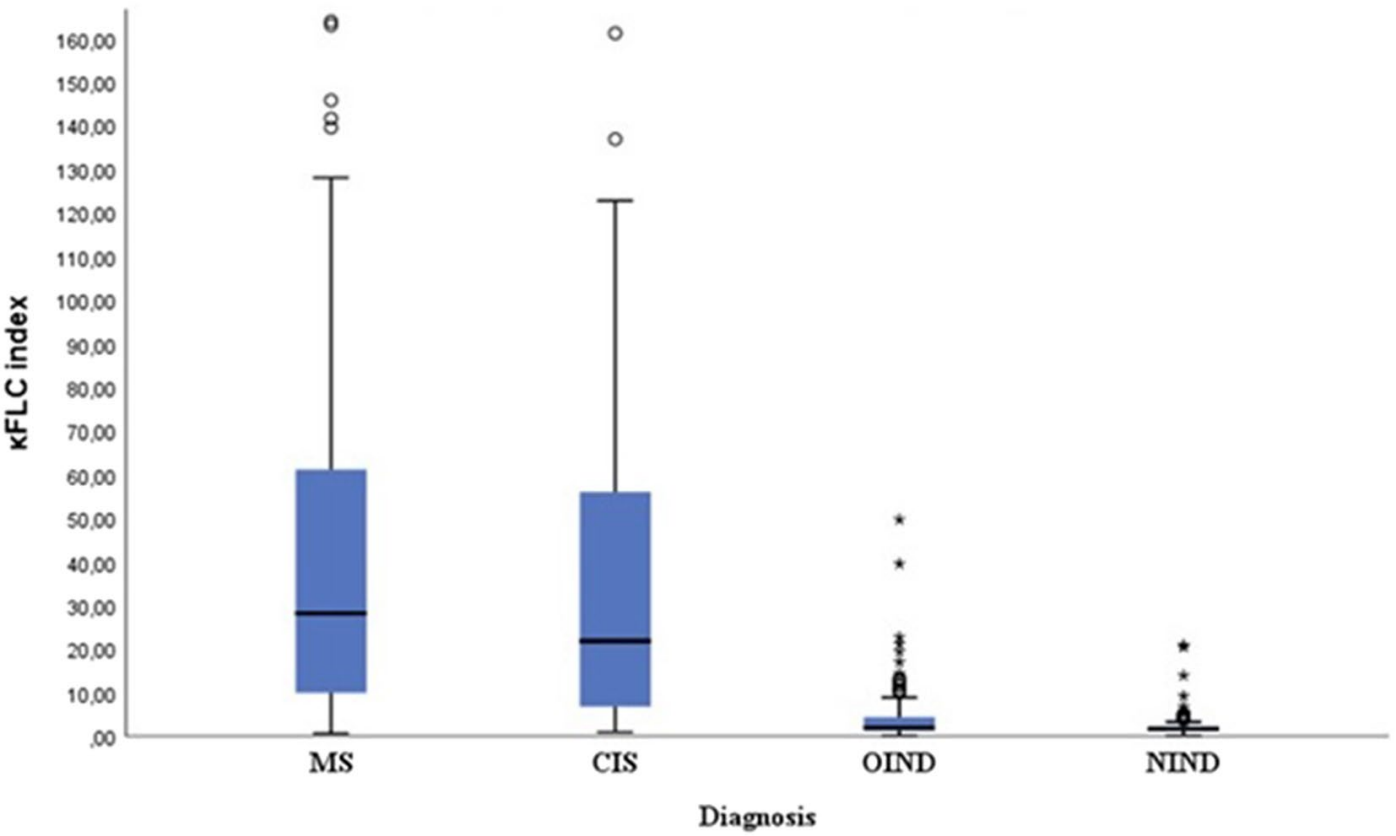
Box plots of κFLC index values according to diagnosis. *κFLC* kappa free light chains, *MS* multiple sclerosis, *CIS* clinically isolated syndrome, *OIND* other inflammatory neurological diseases, *NIND* non-inflammatory neurological diseases

We analyzed paired CSF and serum samples of 607 patients (Table 1, Fig. 1). Among them, 179 patients were diagnosed with MS and 116 with CIS, while 94 and 218 patients were, respectively, affected by OIND and NIND. Patients with MS and CIS were considered together as cases; while, those diagnosed with OIND and NIND were comprehensively considered as controls (Table 2).

**Table 1:** Characteristics of the study population and results from cerebrospinal fluid analysis (607 patients)

	MS	CIS	OIND	NIND
*N*	179	116	94	218
Female *N* (%)	107 (59.8)	85 (72.4)	52 (55.3)	121 (55.5)
Age at diagnosis mean ± SD	40.0 ± 13.1	38.9 ± 14.3	43.1 ± 11.2	61.3 ± 9.8
IgG OCB *N* (%)	141 (78.8)	72 (62.1)	12 (12.8)	3 (1.4)
IgG index (median, IQR)	0.68 (0.56–0.88)	0.59 (0.51–0.84)	0.52 (0.47–0.60)	0.48 (0.44–0.51)
κFLC index (median, IQR)	28.19 (9.81–61.52)	21.84 (6.52–56.19)	1.94 (1.37–4.63)	1.67 (1.36–2.28)

*MS* multiple sclerosis, *CIS* clinically isolated syndrome, *OIND* other inflammatory neurological diseases, *NIND* not inflammatory neurological diseases, *OCB* oligoclonal bands, *IQR* interquartile range, *κFLC* kappa free light chains

**Table 2:** Diagnosis of patients with NIND and OIND

Diagnosis	*N*
**NIND**	**218**
CVD	69
Headache	20
Compressive myelopathy	22
Epilepsy	6
Neurodegenerative	44
Noninflammatory neuropathies	28
Psychogenic	14
Aspecific sensory symptoms	15

Diagnosis	*N*
**OIND**	**94**
NMOSD	14
Inflammatory neuropathies	36
Autoimmune encephalitis	3
Infectious encephalitis	6
Infectious myelopathies	13
Other inflammatory diseases	22

Bold values indicate the total number of patients for NIND (this group amounts to 218 patients and include the underlying categories in the table: CVD, Headache etc) and OIND (this group amounts to 94 patients and include the categories below: NMOSD, Inflammatory neuropathies, etc.)

*NIND* not inflammatory neurological diseases, *CVD* cerebrovascular diseases, *OIND* other inflammatory neurological diseases, *NMOSD* neuromyelitis optica spectrum disorder

### Diagnostic accuracy of CSF IgG OCB and IgG index for the diagnosis of MS/CIS

Among a population of 607 patients (295 MS/CIS, 312 controls), 228 (37.6%) exhibited the presence of CSF IgG OCB. IgG OCB were positive in 213 MS/CIS patients (72.2%) and only in 15 controls (4.8%) (*χ*^2^ = 293.7, *p* < 0.001). Notably, 82 out of 295 MS/CIS patients (27.8%) were OCB negative.

CSF IgG OCB showed 72.2% Se (CI 95% 68.4–75.7) and 95.2% Sp (CI 95% 93.1–96.7) in discriminating between MS/CIS and controls, with PPV of 93.4% (CI 95% 91.1–95.2) and NPV of 78.4% (CI 95% 74.8–81.5) (Table 3). The diagnostic accuracy of CSF IgG OCB was defined by an AUC of 0.84 (CI 95% 0.80–0.87) and by *J* = 0.67.

**Table 3:** Diagnostic performance of different thresholds of κFLC index and IgG OCB for the diagnosis of MS/CIS in our study population (607 patients)

	Se, % (95% CI)	Sp, % (95% CI)	PPV, % (95% CI)	NPV, % (95% CI)	*J*	AUC (95% CI)
4.25	86.8 (83.8–89.3)	88.1 (85.2–90.6)	87.4 (84.4–89.9)	87.6 (84.6–90.0)	0.75	0.875 (0.844–0.905)
5.00	85.4 (82.3–88.1)	90.4 (87.7–92.6)	89.4 (86.6–91.6)	86.8 (83.8–89.3)	0.76	0.879 (0.849–0.909)
5.90	82.0 (78.7–85.0)	92.0 (89.5–94.0)	90.6 (88.0–92.8)	84.4 (81.2–87.2)	0.74	0.870 (0.839–0.901)
6.60	80.0 (76.5–83.1)	92.9 (90.5–94.8)	91.5 (88.9–93.5)	83.1 (79.8–85.9)	0.73	0.865 (0.833–0.869)
7.83	78.0 (74.4–81.2)	93.6 (91.3–95.3)	92.0 (89.5–94.0)	81.8 (78.4–84.7)	0.72	0.858 (0.825–0.890)
10.5	71.9 (67.4–76.0)	94.9 (92.7–96.5)	93.0 (90.6–94.8)	78.1 (74.6–81.3)	0.67	0.834 (0.799–0.868)
11.0	73.2 (68.3–77.7)	95.5 (93.1–97.1)	90.3 (87.3–92.7)	86.1 (82.7–89.0)	0.69	0.832 (0.797–0.866)
12.3	69.2 (65.3–72.8)	96.2 (94.2–97.5)	94.4 (92.2–96.1)	76.7 (73.1–80.0)	0.65	0.827 (0.791–0.862)
IgG OCB	72.2 (68.4–75.7)	95.2 (93.1–96.7)	93.4 (91.1–95.2)	78.4 (74.8–81.5)	0.67	0.84 (0.80–0.87)

*κFLC* kappa free light chains, *OCB* oligoclonal bands, *MS* multiple sclerosis, *CIS* clinically isolated syndrome, *Se* sensitivity, *Sp* specificity, *VPP* positive predictive value, *NPV* negative predictive value, *J* Youden’s index, *AUC* area under the curve, *CI* confidence interval

IgG index values in MS/CIS patients (median = 0.65, IQR = 0.53–0.87) were significantly higher than in controls (median = 0.49, IQR = 0.45–0.54) (*p* < 0.001).

IgG index exhibited 44.4% Se (CI 95% 38.5–50.4) and 95.2% Sp (CI 95% 93.1–96.7), with PPV of 89.7% (CI 95% 87.0–92.0) and NPV of 64.4% (CI 95% 60.5–68.2) for the diagnosis of MS/CIS. The AUC was equal to 0.70 (CI 95% 0.66–0.74) and *J *= 0.39. There was a moderate positive correlation between IgG index and IgG OCB (*r*_pb_ = 0.53, *n* = 607, *p* < 0.001).

The odds of being diagnosed with MS/CIS was fivefold increased (OR = 5.04; 95% CI 2.41–10.56; *p* < 0.001) when IgG OCB were detected; while, IgG index was not a significant risk predictor for the same outcome.

### Diagnostic accuracy of κFLC index for the diagnosis of MS/CIS

**Fig. 2:**
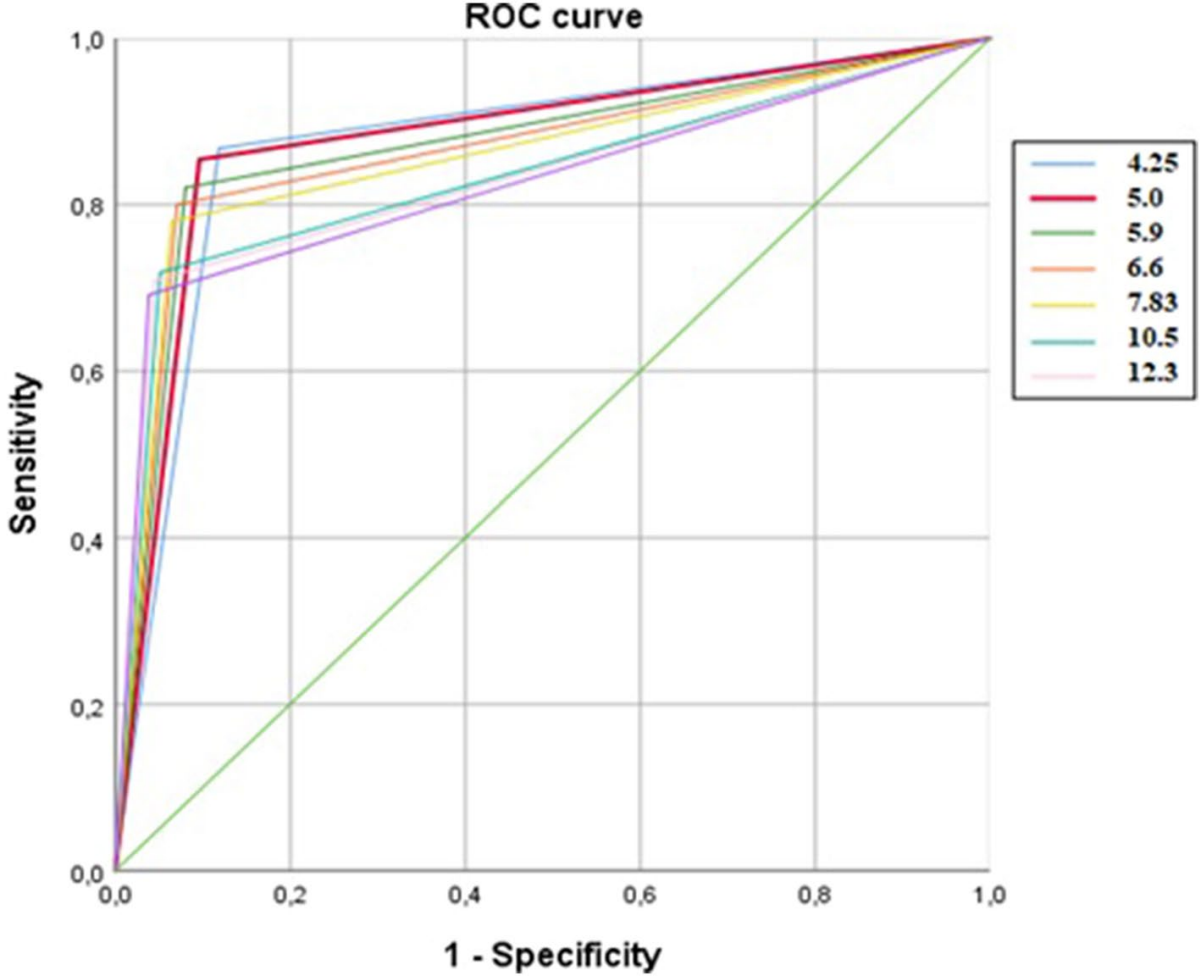
ROC curves for different potential thresholds of κFLC index

κFLC index in MS/CIS patients (median = 26.3, IQR = 9.1–59.5) was significantly higher than in controls (median = 1.7, IQR = 1.4–2.5) (*p* < 0.001). Measures of diagnostic accuracy for different κFLC index thresholds proposed in literature and ROC curves are reported in Table 3 and Fig. 2. Among different thresholds proposed in literature, the cut-off value of 5.0 emerged as the one which maximized the AUC (0.879, CI 95% 0.849–0.909) and the *J* (0.75) in our study population (Table 3). Se and Sp were, respectively, 85.4% (CI 95% 82.3–88.1) and 90.4% (CI 95% 87.7–92.6), with PPV of 89.4% (CI 95% 86.6–91.6) and NPV of 86.8% (CI 95% 83.8–89.3). κFLC index > 5.0 was detected in 43 out of 82 (52.4%) OCB-negative and in 209 out of 213 (98.1%) OCB-positive patients with MS/CIS. Among all proposed thresholds, κFLC index specificity exceeded that of other diagnostic biomarkers for a cut-off of 11.0 (Sp = 95.5%, CI 95% 93.1–97.1), and PPV peaked to 90.3% (CI 95% 87.3–92.7), though reducing Se (73.2%, CI 95% 68.3–77.7) and NPV (86.1%, CI 95% 82.7–89.0).

AUCs for all thresholds between 4.25 and 6.6 were higher than the AUCs of cut-off ≥ 10.5, while they were not significantly different from each other (Table 4). The interval of κFLC index values between 4.25 and 6.6 was characterized by Se values between 80.0 and 86.8%, and Sp between 88.1 and 92.9% (Table 3). AUCs for thresholds between 4.25 and 6.6 were significantly higher than the AUC of IgG OCB (Table 5). Positive κFLC index values, according to the chosen threshold between 4.25 and 6.6, were detected in 37.8–54.9% of OCB-negative patients with MS/CIS.

**Table 4:** Paired comparison between AUCs of different κFLC index thresholds for the diagnosis of MS/CIS in our study population (607 patients)

κFLC index	*z*	*p*	Delta AUC	95% CI	
				Lower limit	Upper limit
4.25–5.0	− 0.824	0.410	− 0.004	− 0.015	0.006
4.25–5.90	0.545	0.586	0.004	− 0.012	0.021
4.25–6.60	1.036	0.300	0.010	− 0.009	0.029
4.25–7.83	1.606	0.108	0.017	− 0.004	0.037
4.25–10.5	3.252	**0.001**	0.041	0.016	0.066
4.25–11.0	3.295	**0.001**	0.043	0.017	0.068
4.25–12.3	3.556	**0.000**	0.048	0.022	0.075
5.0–5.90	1.404	0.160	0.009	− 0.004	0.021
5.0–6.60	1.791	0.073	0.014	− 0.001	0.030
5.0–7.83	2.325	**0.020**	0.021	0.003	0.039
5.0–10.5	3.917	**0.000**	0.045	0.023	0.068
5.0–11.0	3.923	**0.000**	0.047	0.024	0.071
5.0–12.3	4.157	**0.000**	0.053	0.028	0.077
5.90–6.60	1.081	0.280	0.005	− 0.004	0.015
5.90–7.83	1.820	0.069	0.012	− 0.001	0.026
5.90–10.5	3.639	**0.000**	0.036	0.017	0.056
5.90–11.0	3.622	**0.000**	0.038	0.018	0.059
5.90–12.3	3.859	**0.000**	0.044	0.021	0.066
6.60–7.83	1.483	0.138	0.007	− 0.002	0.016
6.60–10.5	3.501	**0.000**	0.031	0.014	0.048
6.60–11.0	3.457	**0.001**	0.033	0.014	0.052
6.60–12.3	3.691	**0.000**	0.038	0.018	0.059
7.83–10.5	3.140	**0.002**	0.024	0.009	0.039
7.83–11.0	3.075	**0.002**	0.026	0.009	0.043
7.83–12.3	3.323	**0.001**	0.031	0.013	0.050
10.5–11.0	0.508	0.611	0.002	− 0.005	0.009
10.5–12.3	1.252	0.211	0.007	− 0.004	0.018
11.0–12.3	1.200	0.230	0.005	− 0.003	0.014

Bold values indicate the statistically significant *p* values

*κFLC* kappa free light chains, *AUC* area under the curve, *CI* confidence interval

**Table 5:** Paired comparison between AUCs of CSF IgG OCB and different κFLC index thresholds for the diagnosis of MS/CIS in our study population (607 patients)

IgG OCB-κFLC	*z*	*p*	Delta AUC	95% CI	
				Lower limit	Upper limit
4.25	− 2.725	**0.006**	− 0.038	− 0.065	− 0.011
5.0	− 3.153	**0.002**	− 0.042	− 0.068	− 0.016
5.90	− 2.656	**0.008**	− 0.033	− 0.058	− 0.009
6.60	− 2.262	**0.024**	− 0.028	− 0.052	− 0.004
7.83	− 1.744	0.081	− 0.021	− 0.044	0.003
10.5	0.263	0.792	0.003	− 0.021	0.028
11.0	0.410	0.682	0.005	− 0.020	0.030
12.3	0.851	0.395	0.010	− 0.014	0.035

Bold values indicate the statistically significant *p* values

*κFLC* kappa free light chains, *AUC* area under the curve, *CI* confidence interval

**Fig. 3:**
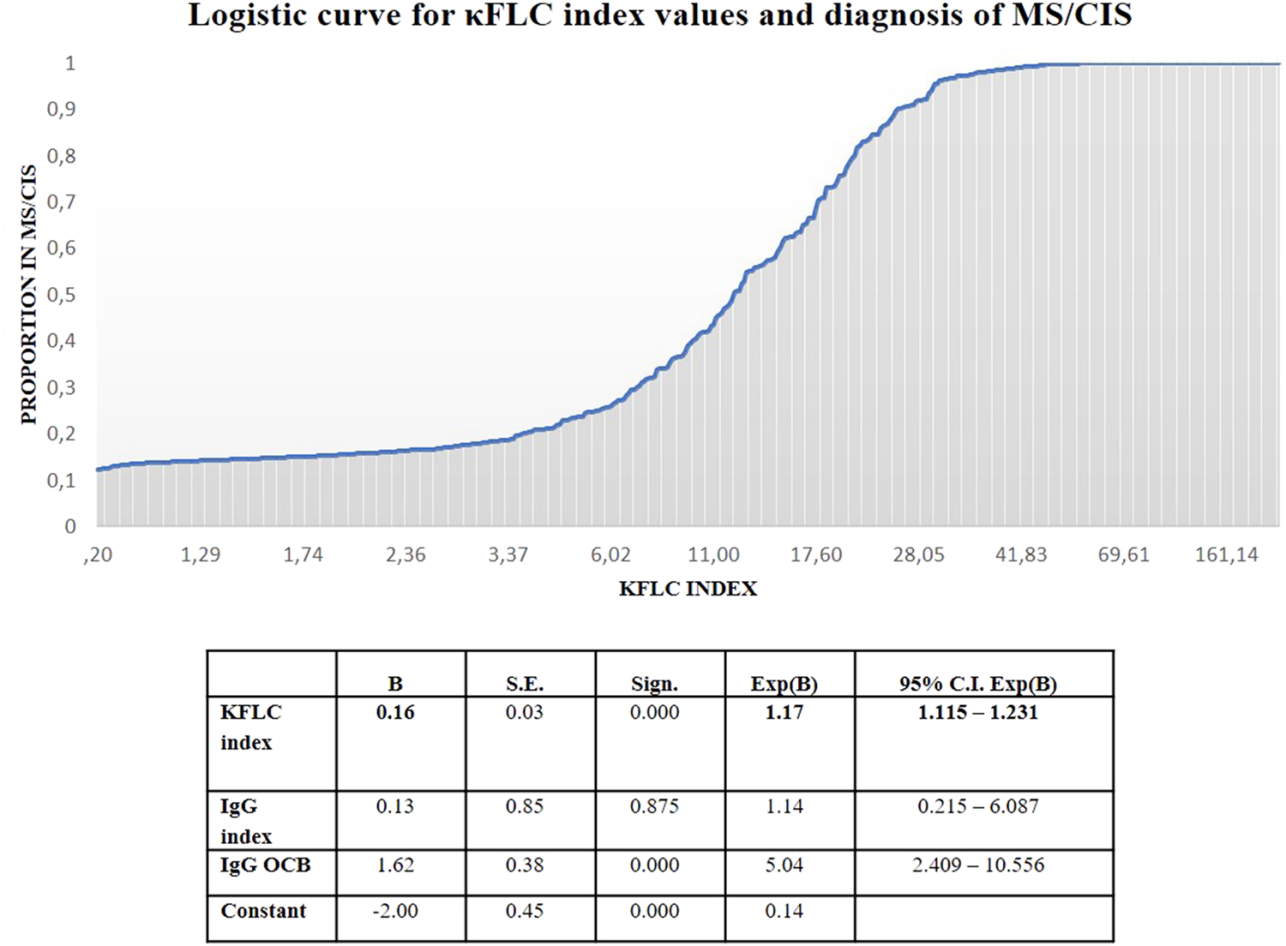
Probability of diagnosis of MS/CIS based on the values of the independent variable KFLC index. *κFLC* kappa free light chains, *MS* multiple sclerosis, *CIS* clinically isolated syndrome, *OCB* oligoclonal bands

The binary logistic regression analysis, even when IgG index and IgG OCB were used as covariates, confirmed that the odds of being diagnosed with MS/CIS increased by 17.1% for each unit increase of κFLC index (OR = 1.17; 95% CI 1.12–1.23; *p* < 0.001) (Fig. 3). For each increase of 5 units in κFLC index values, OR is expected to increase by 2.2 times [OR = (1.17)^5^].

## Discussion

CSF IgG OCB detection has generally been considered the gold standard to assess intrathecal synthesis in patients with MS and its introduction in the latest revision of McDonald’s criteria as a substitute for dissemination in time (DIT) has further enhanced its diagnostic role[4]. This recent acquisition highlighted even more the importance of performing CSF collection and analysis, already implemented in clinical practice, in patients suspected with MS. In our analysis, CSF IgG OCB showed Se of 72.2% and Sp of 95.2% in distinguishing between patients diagnosed with MS/CIS and patients with other neurological diseases, regardless of their inflammatory or not inflammatory nature. This is in agreement with a high number of results from previous studies, which reported for IgG OCB sensitivity values ranging from 83 and 95% [3, 20, 22–24] and Sp ranging from 86 to 95% [3, 20, 25] for the diagnosis of MS. Further, we detected CSF IgG OCB in 78.8% of MS patients and 62.1% of CIS, values similar to those found by Dobson and co-workers in a large meta-analysis of 71 articles, involving more than 12,000 patients with MS (87.7% of MS, 68.6% of CIS) [22].

An IgG index higher than 0.7 was detected in 49.2% of our MS subgroup and in 37.1% of CIS, roughly in line with previous literature data reporting values between 50 and 75% [19, 26]. Furthermore, it showed good Sp in our analysis (95.2%) when comparing MS/CIS with other neurological diseases, but very low Se (44.4%). Other studies reported good Sp for IgG index, together with a good concordance with the detection of CSF IgG OCB [18, 19]. Differently, the correlation between IgG index and IgG OCB was only moderate in our analysis.

κFLC index showed a higher sensitivity than CSF IgG OCB in all comparisons. When distinguishing patients with MS/CIS from controls, by choosing a threshold of 5.0, κFLC index showed a sensitivity of 85.4% (vs 72.2% of CSF IgG OCB), NPV of 86.8% (vs 78.4% of CSF IgG OCB) and good specificity and PPV, despite lower than values reported for CSF IgG OCB (90.4% vs 95.2% and 89.4% vs 93.4%, respectively). Other studies reported a higher sensitivity of κFLC index compared with CSF IgG OCB, but a lower specificity, as in our analysis [3, 11]. However, this result is not univocal and the lack of an established cut-off may limit the comparison among literature data [27, 28] (Table 6).

**Table 6:** Sensitivity and specificity values for different thresholds of κFLC index reported in previous studies and characteristics of the study cohorts

Cut-off	Sensitivity	Specificity	Patients	Cases	McDonald’s criteria	
≥ 4.25	94%	100%	137	MS (70)	2017	Puthenparampil et al. (2018)
≥ 5	96%	78%	385	MS (127)	2017	Crespi et al. (2019)
≥ 5.9	96%	86%	438	CIS/MS (70)	2010	Presslauer et al. (2016)
≥ 6.6	93%	83%	745	CIS, MS (526)	2010	Leurs et al. (2019)
≥ 7.83	89%	81%	170	RIS, CIS, MS (64)	2010	Gaetani et al. (2020)
≥ 10.5	87%	76%	320	RIS, CIS, MS (67)	2010	Gurtner et al. (2018)
≥ 12.3	93%	100%	176	MS (71)	2010	Pieri et al. (2017)

*MS* multiple sclerosis, *CIS* clinically isolated syndrome, *RIS* radiologically isolated syndrome

Compared with a cut-off value of 5.0, which maximized the AUC (0.879, CI 95% 0.849–0.909) and J index (0.75), thresholds higher than 5 (5.9 [10], 6.6 [11], 7.83 [3], 10.5 [9], 12.3 [6]) showed higher specificity but lower sensitivity in our study cohort, with generally lower AUC and J. Of note, as shown in Table 3, different κFLC index potential cut-off values explored in our analysis exhibited AUCs higher than the one of OCB (0.84), but all have lower values for Sp, as found in other studies [14].

The threshold of 4.23 suggested by Putheranpamil and co-workers [7] showed slightly increased sensitivity and decreased specificity in our sample, with lower J index and similar AUC. Moreover, Crespi and co-workers [29] identified the same threshold of 5.0 chosen in our study, though finding different sensitivity and specificity values (96% vs our 85.4% and 78% vs our 90.4%, respectively).

Comparisons among different studies are certainly limited by several factors. First, different revisions of McDonald’s criteria were used by different authors and patients with CIS have not always been considered together with MS as “cases” (Table 6). Second, the use of different commercial assays to detect κFLC in CSF and serum in different laboratories can hamper the repeatability of results. This could be also due to the different protein sources adopted by different commercial suppliers and therefore also by different laboratories. To partially overcome these limitations, we tested and applied all the thresholds proposed in literature in our study population, recruited according to the latest revision of McDonald’s the same criteria and tested with a unique technical procedure, including the use of the same monoclonal antibodies and dilutions of test samples. However, other potential sources of error include the underestimation or overestimation of FLC concentrations due to antigen excess and polymerization effects [30]. On the one hand, this could be a further stimulus to overcome the concept of choosing a unique threshold and consider a more “dynamic” interpretation of κFLC index. On the other hand, since extensive data have been provided so far from several studies on quite similar cut-off values for κFLC index without conclusive results, multicenter studies using different platforms and assays should be performed to definitively confirm these thresholds, and certified reference materials should be developed.

As expected, patients with MS/CIS exhibited significantly higher κFLC index values than controls. Se values between 80.0 and 86.8% and Sp between 88.1 and 92.9% were reported for κFLC index interval 4.25–6.6, with no significant differences in the AUCs of the explored thresholds 4.25, 5.0, 5.9, 6.6. Based on our results, this prevents in fact to assert that one cut-off value is superior to another for values between 4.25 and 6.6, suggesting that the lack of a univocal cut-off, which is currently the main limitation for the use of κFLC index in clinical practice, is not an insurmountable problem. Further, κFLC index AUC was higher than IgG OCB AUC when considering thresholds between 4.25 and 6.6, while no differences emerged for values ≥ 7.83. Therefore, we should take in account that IgG OCB exhibit a lower or at least equal diagnostic accuracy compared with κFLC index.

Several previous studies reported a higher Se of κFLC index compared with CSF IgG OCB, but a lower Sp [3, 11]. To overcome this issue, Gaetani and colleagues suggested the choice of a higher κFLC index cut-off when discriminating between MS/CIS and OIND, in order to increase Sp [3]. However, these results are not univocal and the lack of an established cut-off has partially limited the comparison among literature data [27, 28] (Table 6). Finally, a recent metanalysis, including results from 32 studies, identified a value of 6.1 as the better discriminatory cut-off, but found no significant differences between κFLC index and IgG OCB in terms of diagnostic accuracy [14].

Evidently, being a quantitative continuous variable, κFLC index exhibits an intrinsic advantage compared with the analysis of IgG OCB, since values are much more informative about the risk of being diagnosed with MS/CIS. As a consequence, the use of IEF could be restricted only to cases actually characterized by elements of uncertainty, including atypical MRI lesions, non-specific symptoms or κFLC index values close to the lower limit of the interval (i.e., values between 4.25 and 6.6).

It is known that IgG OCB are currently the gold standard as a biomarker of intrathecal synthesis in MS and that their detection can substitute for DIT according to the 2017 revision of McDonald’s criteria [4], actually limiting the use of other diagnostic biomarkers for MS. Further, this limitation also relies on the fact that quantitative determinations (e.g., IgG index, κFLC index) are less reliable than qualitative ones, since they depend on the specificity of the antiserum used and are more subject to variability of results among laboratories [31].

However, κFLC index reflects the intrathecal synthesis of CSF κFLC, which are produced in excess during the synthesis of Ig, consequently sharing the same physiopathological substrate with OCB. If technical limitations were exceeded, κFLC index could then represent a valuable instrument to substitute for DIT, or to support the diagnosis of MS in OCB-negative patients or when DIS and DIT are already satisfied by clinical and radiological criteria. It might be interesting to evaluate OCB-negative CIS patients with high κFLC index values over time, to assess whether they might benefit of an earlier diagnosis of MS, with consequent therapeutic implications, assuming κFLC index as a substitute for DIT.

If the identification of a threshold is important to exclude the diagnostic suspicion in controls, the increase in the risk of being diagnosed with MS/CIS along with the increase of κFLC index values is even more crucial. Indeed, evidence from clinical practice confirm that lots of patients diagnosed with MS exhibit very high κFLC index values, much higher than the possible cut-off explored, and that they are more likely to be diagnosed with MS/CIS. However, this observation would have no specific meaning when a dichotomous interpretation of κFLC index is used.

In our population, each increase of 5 units in κFLC index value corresponded to a 2.2-fold higher risk of being diagnosed with MS/CIS. In other words, for progressively increasing κFLC index values, the probability of being diagnosed with MS/CIS can be represented by an exponential curve (Fig. 3).

Based on our findings, κFLC index is not only highly sensitive in excluding a diagnosis of MS and precursory conditions during the diagnostic workout, but also exhibits the irreplaceable advantage of being a quantitative variable, which lends itself to a flexible interpretation. Additionally, it is notably less time-consuming and less expensive than OCB analysis. It has been estimated that the cost of IEF for the detection of IgG OCB amounts to 23.5 euros/patient (including materials, controls, antisera), which adds to personnel cost (about 15 euros/hour), for a total of approximately 46 euros/patient. Further, three working hours are required to evaluate IgG OCB in CSF of two patients [32]. Differently, about 16 euros/patient for material costs are required for the analysis of κFLC index and only 10 min are needed for evaluating two patients, thus significantly reducing personnel cost as well (for a total of about 17.25 euros/patient). Consequently, the exclusive use of κFLC index for diagnostic purpose would have saved about 62.5% of costs and have taken about 18 times less than the analysis of IgG OCB for the entire study population, in line with data reported by Crespi and colleagues [32]. Indeed, the analysis of CSF IgG OCB implies a costly multistep method requiring paired CSF and serum specimens to be run in parallel, with a subjective visual interpretation, and an average time for the analytical processing of over 3 h. Moreover, IEF is a qualitative assessment and there is no standard definition of the IgG OCB amounts required for a clinically positive result (anything from 1 to 4 unique CSF bands). In this regard, package inserts suggest establishing an individual laboratory reference interval within its own population, despite the FDA approval of IEF testing [9].

**Fig. 4:**
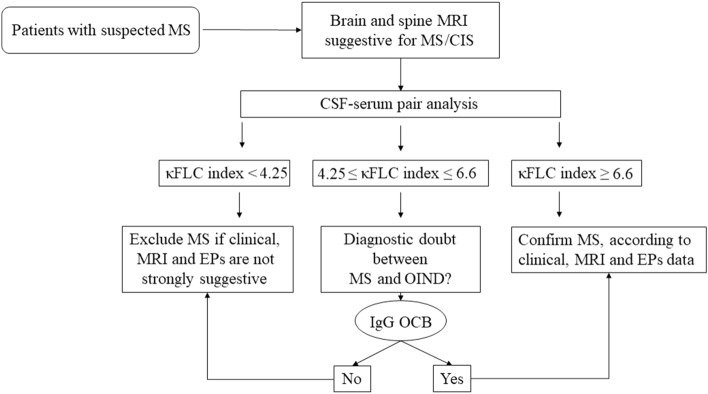
Procedural algorithm for the diagnosis of multiple sclerosis. *MS* multiple sclerosis, *MRI* magnetic resonance imaging, *CSF* cerebrospinal fluid, *κFLC* kappa free light chains, *OIND* other inflammatory neurological diseases, *EPs* evoked potentials

Comprehensively, we propose to use κFLC index as a preliminary test, which can be useful not only to exclude the diagnosis of MS/CIS in the appropriate clinical context when values below the considered range are detected, but also to predict the probability of MS/CIS diagnosis with greater confidence the higher κFLC index values. The use of IgG OCB, which currently remains the gold standard for the diagnosis of MS, could be restricted to patients with κFLC index values between 4.25 and 6.6 or according to clinical judgement, to provide further confirmation in doubtful cases (Fig. 4). Additionally, the analysis of CSF IgG OCB should be performed when DIT cannot be provided otherwise, according to the latest revision of McDonald’s criteria.

It should also be noted that κFLC index can correctly identify OCB-negative MS and CIS patients, who amounted to 21.2% and 37.9%, respectively, in our sample. Particularly, 37.8–54.9% of OCB-negative MS/CIS patients exhibited positive κFLC index values in our study, according to the chosen thresholds between 4.25 and 6.6. This was quite in line with data reported by Ferraro and co-workers in a recent study, showing that a κFLC index ≥ 5.8 was detected in 25% of OCB-negative MS patients and in 98% of OCB-positive ones [33].

Based on our results, the use of κFLC index in clinical practice could be highly beneficial, providing an easily and quickly achieved, cost-effective and helpful support for the diagnosis of MS, leading itself to a flexible interpretation in the appropriate clinical context.

## Data Availability

The datasets analyzed during the current study are available from the corresponding author on reasonable request.
